# Incidents from Practice

**Published:** 1904-01

**Authors:** 


### INCIDENTS FROM PRACTICE.

Dr. Russell: It is not often that attention is called in societies to the necessity of more care by the dentist, in regard to the hands and instruments. A case of infection that I recently had to trace up has proved to my mind that we all ought to be more careful than we are. The case was that of a young, healthy woman about twenty-five years of age, who was in the country—not exposed to infection of any kind. She went to a dentist, and had some teeth filled. This dentist was a consumptive in pretty bad shape, and a few days after he finished his work, her mouth became very sore and her teeth loose, and three weeks afterwards she had a hard cough, and rapidly developed all symptoms of consumption. Examination of the sputum and the lungs showed tuberculosis bacilli in the saliva, and a consolidation in the lung. It is generally difficult to trace the case to the man who is responsible for it; but to my mind there are many cases where infection is given by the dentist through his hands or instruments. I think the dentist's hands are often responsible. They do not clean their hands properly. I dare say among the members of this society you will not find a bi-chloride solution in one office out of fifty; and yet dentists handle consumptive cases, and go from one to the other, just washing their hands with soap and water. It is not fair to the patient, and I think it ought to be brought more fully before the dentists.

Dr. Walker: Would Dr. Rusesll recommend using the solution of bi-chloride after each patient? What would be the condition of the hands?

Dr. Russell: If you use alcohol and glycerine between the washings, you can have your hands soft and nice at the end of the day. Sometimes a man will wash his hands ten times in an hour, and yet he will not have his hands very rough. We use it down at the Polhemus Clinic.

Dr. Hutchinson: I have a little incident to report, that may prove interesting. dent to report, that may prove interesting. A patient who was in the habit of coming to me quite frequently for examination, came to my office a couple of months ago with the left superior cuspid showing a pinkish tinge throughout the entire labial surface. I found there was an exposure of soft living tissue. She had been in my office only a short time previously and there was no sign of anything peculiar. I anæsthetized the mouth with chloride of ethyl, and introduced an excavator to see whether it was a fungus growth, or whether it was from the pulp, and I found it was from the pulp. The tooth broke off, and I finally removed the pulp. The tooth had previously been filled with cement to an extreme depth—a depth that would indicate in most cases an exposure of pulp, and we would expect it to be followed by the death of the pulp; but in this case the pulp had survived and enlarged, and resorption of the dentine and enamel had taken place, until the pulp had exposed itself on the surface.

